# Exosomes derived from human umbilical cord mesenchymal stem cells protect against cisplatin-induced ovarian granulosa cell stress and apoptosis *in vitro*

**DOI:** 10.1038/s41598-017-02786-x

**Published:** 2017-05-31

**Authors:** Liping Sun, Dong Li, Kun Song, Jianlu Wei, Shu Yao, Zhao Li, Xuantao Su, Xiuli Ju, Lan Chao, Xiaohui Deng, Beihua Kong, Li Li

**Affiliations:** 1 Department of obstetrics and gynecology, Qilu Hospital, Shandong University, 107 Wenhua Xi Road, Jinan, 250012 Shandong Province P.R. China; 2 Cryomedicine Laboratory, Qilu Hospital, Shandong University, 107 Wenhua Xi Road, Jinan, 250012 Shandong Province P.R. China; 3 Department of Orthopedics, Qilu Hospital, Shandong University, 107 Wenhua Xi Road, Jinan, 250012 Shandong Province P.R. China; 4 Department of obstetrics and gynecology, Qianfoshan Hospital, Shandong University, 16766 Jingshi Road, Jinan, 250014 Shandong Province P.R. China; 5 Institute of Biomedical Engineering, School of Control Science and Engineering, Shandong University, 17923 Jingshi Road, Jinan, Shandong 250061 P.R. China; 6 Reproduction Medicine Center, Qilu Hospital, Shandong University, 107 Wenhua Xi Road, Jinan, 250012 Shandong Province P.R. China

## Abstract

Human umbilical cord mesenchymal stem cells (huMSCs) can treat primary ovarian insufficiency (POI) related to ovarian granulosa cell (OGC) apoptosis caused by cisplatin chemotherapy. Exosomes are a class of membranous vesicles with diameters of 30–200 nm that are constitutively released by eukaryotic cells. Exosomes mediate local cell-to-cell communication by transferring microRNAs and proteins. In the present study, we demonstrated the effects of exosomes derived from huMSCs (huMSC-EXOs) on a cisplatin-induced OGC model *in vitro* and discussed the preliminary mechanisms involved in these effects. We successfully extracted huMSC-EXOs from huMSC culture supernatant and observed the effective uptake of exosomes by cells with fluorescent staining. Using flow cytometry (with annexin-V/PI labelling), we found that huMSC-EXOs increased the number of living cells. Western blotting showed that the expression of Bcl-2 and caspase-3 were upregulated, whilst the expression of Bax, cleaved caspase-3 and cleaved PARP were downregulated to protect OGCs. These results suggest that huMSC-EXOs can be used to prevent and treat chemotherapy-induced OGC apoptosis *in vitro*. Therefore, this work provides insight and further evidence of stem cell function and indicates that huMSC-EXOs protect OGCs from cisplatin-induced injury *in vitro*.

## Introduction

With the increased incidence of gynaecological cancer, clinical application of chemical treatment is prevalent. Chemotherapy drugs can significantly reduce the number of follicles in ovarian tissues and damage ovarian stroma, causing endocrine disorders and reproductive dysfunction or primary ovarian insufficiency (POI), which lead to symptoms such as menstrual disorders, amenorrhea and infertility^1^. Several studies have shown that POI has a close relationship with the injury and apoptosis of ovarian granulosa cells (OGCs). Hence, the reproductive toxicity caused by chemotherapy is of great concern, particularly in young and fertile female patients.

Mesenchymal stem cells (MSCs) derived from the early development of the mesoderm and ectoderm are an important member of the adult stem cell family^2^. MSCs were originally found in bone marrow, and studies have shown that bone marrow MSC transplantation can be used to treat ischaemia and repair damaged tissues^3, 4^. Thereafter, previous studies have indicated that a large number of MSCs exist in umbilical cord tissues, known as human umbilical cord mesenchymal stem cells (huMSCs)^5^. Clinical application of huMSCs is extensive, because not only do huMSCs express all the biological characteristics of bone marrow MSCs but they also exhibit good proliferation, differentiation potential and low immunogenicity^6^. Therefore, huMSCs are ideal seed cells in tissue engineering^7^. Furthermore, numerous studies have explored huMSC treatments and the effect on various conditions, such as acute lung injury, diabetes with insulin resistance and Alzheimer’s disease^8–11^.

Studies have shown that the application of stem cells can achieve follicle regeneration^12^. Lee *et al*. confirmed that after MSC transplantation, which can significantly increase the number of ovarian follicles and the oestradiol concentration, mice with chemotherapy-induced POI can maintain long-term fertility, suggesting that MSCs can help repair the ovary structure and improve ovarian function^13^. These effects may be closely related to the suppression of OGC apoptosis. Furthermore, other studies have found that huMSCs, secreting several factors associated with the growth and development of tissues, are involved in injury repair and can be induced to differentiate bone cells, nerve cells, heart cells^14^. MSCs directly migrate to the damaged ovaries and differentiate into follicular cells in the ovarian microenvironment to promote the recovery of reproductive endocrine function and inhibit OGC apoptosis^15^. However, the mechanisms responsible for these changes are not entirely clear.

In recent years, the functions of exosomes have been widely studied. Exosomes are a class of membranous vesicles that are 30–200 nm in diameter and are natural nanoparticles secreted by mammalian cells. Exosomes are widely present in different biological fluids and contain mRNA, long noncoding RNA (lncRNA), microRNAs, proteins and lipids^16, 17^. Exosomes transfer these contents to mediate communication between cells and modify genes or proteins in target cells^18^. For example, studies have shown that dendritic cell (DC)-derived exosomes may target and activate CD4 (+) T cells through the endocrine pathway to improve cardiac function after myocardial infarction^19–21^.

A multi-chambered vesicle, termed a multivesicular body, is formed by the retraction of the phospholipid bilayer of the host cells. Multivesicular bodies are bound to the cell membrane in a calcium-dependent manner and release extracellular vesicles known as exosomes. Exosomes can be absorbed via several mechanisms, including membrane fusion, endocytosis and binding to cell surface receptors^22^. The exosome surface contains many biomarkers, such as annexins, Rabs, TSG101, CD63, CD81, CD9, ALIX and Hsp70. In addition, exosomes can also carry specific oncogenic proteins expressed by cancer cells, such as EpCAM and EGFR^23–25^. However, exosomes do not contain the endoplasmic reticulum marker calnexin or the lysosome marker lysosomal-associated membrane protein 1 (Lamp 1).

Exosomes, which are important messengers between cells, regulate other cells. Numerous studies have been conducted the biological effects of exosomes secreted by tumour and stem cells. Wang *et al*. noted that exosomes derived from MSCs promote the proliferation, survival and drug resistance of multiple myeloma cells^26^. Studies have noted that exosomes from stem cells play a role in wound healing. Zhang *et al*. reported that huMSC-EXOs play a positive role in skin wound healing via the Wnt-4 signalling pathway^27^. Several studies have shown that huMSCs have positive effects on chemotherapy-induced POI. However, the mechanisms behind these effects are not clear. In the present study, we examined the effects of huMSC-EXOs on a cisplatin-induced OGC model *in vitro* and determined the preliminary mechanisms.

## Results

### Typical characteristics of huMSCs

**Figure 1:**
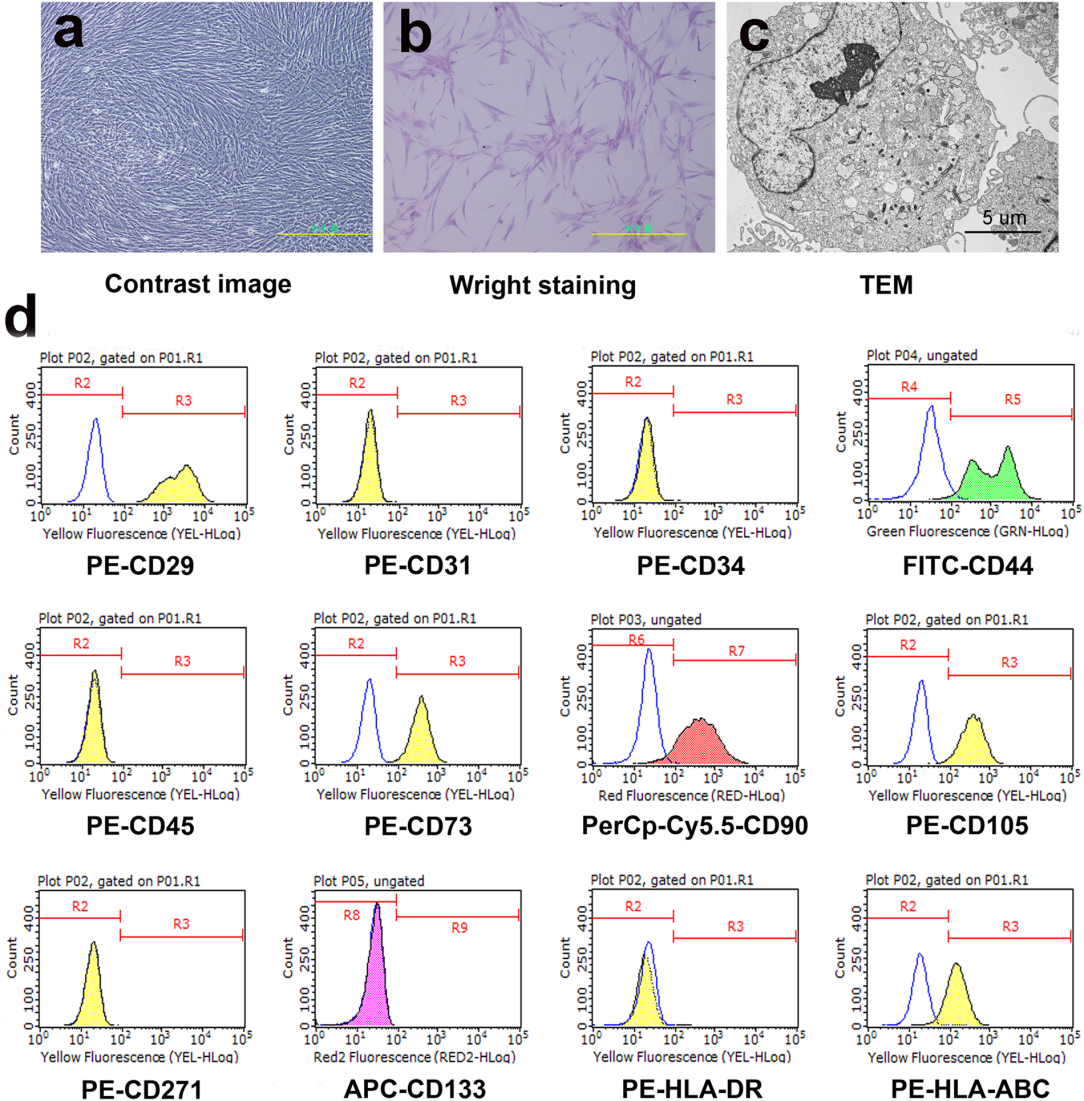
Typical characteristics of huMSCs. (**a**,**b**) HuMSC morphology was polygonal, swirling and fibroblast-like (×100 magnification). (**b**) Wright staining. (**c**) TEM showed that the connection between huMSCs primarily depended on microvilli contact, whilst tight junctions were occasionally visible. (**d**) HuMSC expression of CD29, CD44, CD73, CD90, CD105 and HLA-ABC was visibly high. However, the expression CD31, CD34, CD45, CD133, CD271 and HLA-DR was negative; n = 5.

We observed that huMSCs were a class of polygonal, swirling and fibroblast-like cells (Fig. 1a and b). Transmission electron microscopy (TEM) showed that connections between the huMSCs primarily depended on microvilli contact, whilst tight junctions were occasionally visible (Fig. 1c). Fluorescence-activated cell sorting (FACS) demonstrated that huMSC markers, including CD29, CD44, CD73, CD90, CD105 and HLA-ABC, were highly expressed. Furthermore, the negative markers CD31, CD34, CD45, CD133, CD271 and HLA-DR were not expressed (Fig. 1d). Therefore, huMSCs obtained by the method described above expressed the typical markers of MSCs; n = 5.

### Typical characteristics of huMSC-EXOs

**Figure 2:**
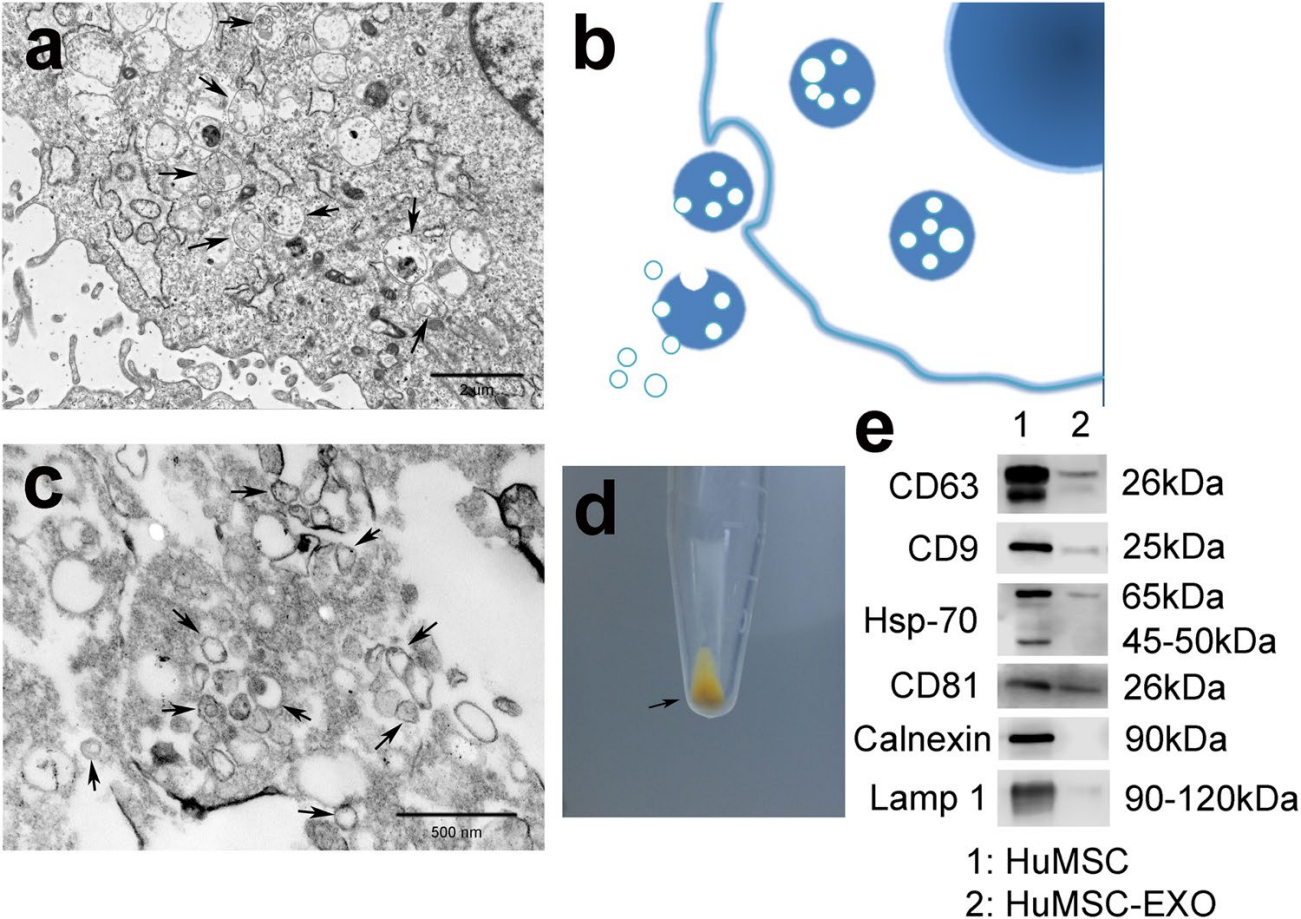
Typical characteristics of huMSC-EXOs. (**a**,**b**) The cellular lipid bilayer is retracted to form multi-chambered vesicles, which release nanoscale vesicles, named exosomes, in a calcium-dependent manner that bind to cell membranes. (**b**) The simulation diagram. (**c**) TEM showed the morphology of exosomes, which were 30–200 nm in diameter. (**d**) The exosomes precipitated in the bottom of the tube, and were a light yellow colour. (**e**) Western blotting analysis indicated that huMSC-EXOs expressed exosomal markers, such as CD63, CD9, Hsp70 and CD81 proteins. However, calnexin and Lamp1 were not expressed; n = 5.

To further obtain huMSC-EXOs, we used gradient ultracentrifugation to extract exosomes from the culture medium. Exosomes precipitated in the bottom of the tube and were light yellow in colour (Fig. 2d). The cellular lipid bilayer retracts to form multi-chambered vesicles, which results in the release of nanoscale vesicles (exosomes) in a calcium-dependent manner that bind to cell membranes. The vesicle-like morphology of exosomes was visualized via TEM, which confirmed exosome diameters of 30 to 200 nm (Fig. 2a,b and c). Fig. 2b was the simulation diagram. Western blotting analysis indicated that huMSC-EXOs expressed exosomal markers, such as CD63, CD9, Hsp70 and CD81 proteins, but did not express the endoplasmic reticulum marker calnexin or the lysosome marker Lamp 1, which showed that huMSC-EXOs isolated by the processes described above did not contain the components or pieces of the endoplasmic reticulum or lysosomes (Fig. 2e). Hence, huMSC-EXOs expressed the typical markers of exosomes and were used in the following experiments; n = 5.

### Characteristics of OGCs and a cisplatin-induced cell model

**Figure 3:**
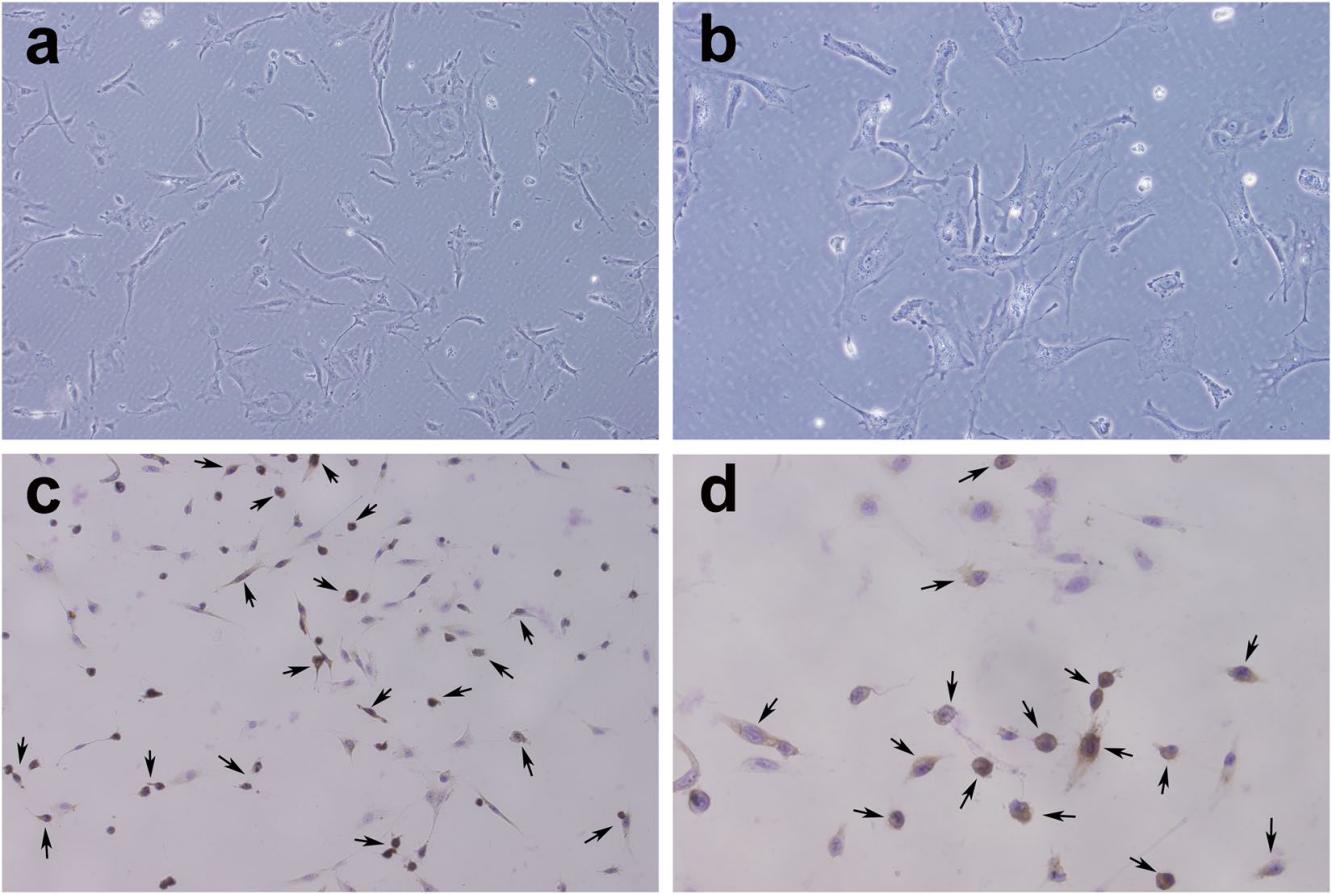
Typical characteristics of OGCs. (**a**,**b**) The OGC morphology was polygonal and fibre-like. (**c**,**d**) After FSHR immunostaining, a large number of adherent cells were dyed with DAB, producing a brown colour, and the dyed cells accounted for approximately 70–80% of adherent cells. Brown cells stained with DAB were OGCs. See arrows (**a** and **c**, ×200 magnification; **b** and **d**, ×400 magnification); n = 5.

The cells were adherent and grew well after 48 h of inoculation, exhibiting polygonal and fibre-like structures (Fig. 3a and b). After follicle-stimulating hormone receptor (FSHR) immunostaining, OGCs were dyed brown with DAB, which accounted for approximately 70–80% of the adherent cells. The brown cells stained with DAB were the OGCs, indicating that OGCs derived from rats were successfully cultured *in vitro*; n = 5 (Fig. 3c and d).

Cisplatin was used in the cell model. Based on our pre-tests, 4 µg/ml was determined to be the optimal experimental concentration.

### Effective uptake of huMSC-EXOs by OGCs

**Figure 4:**
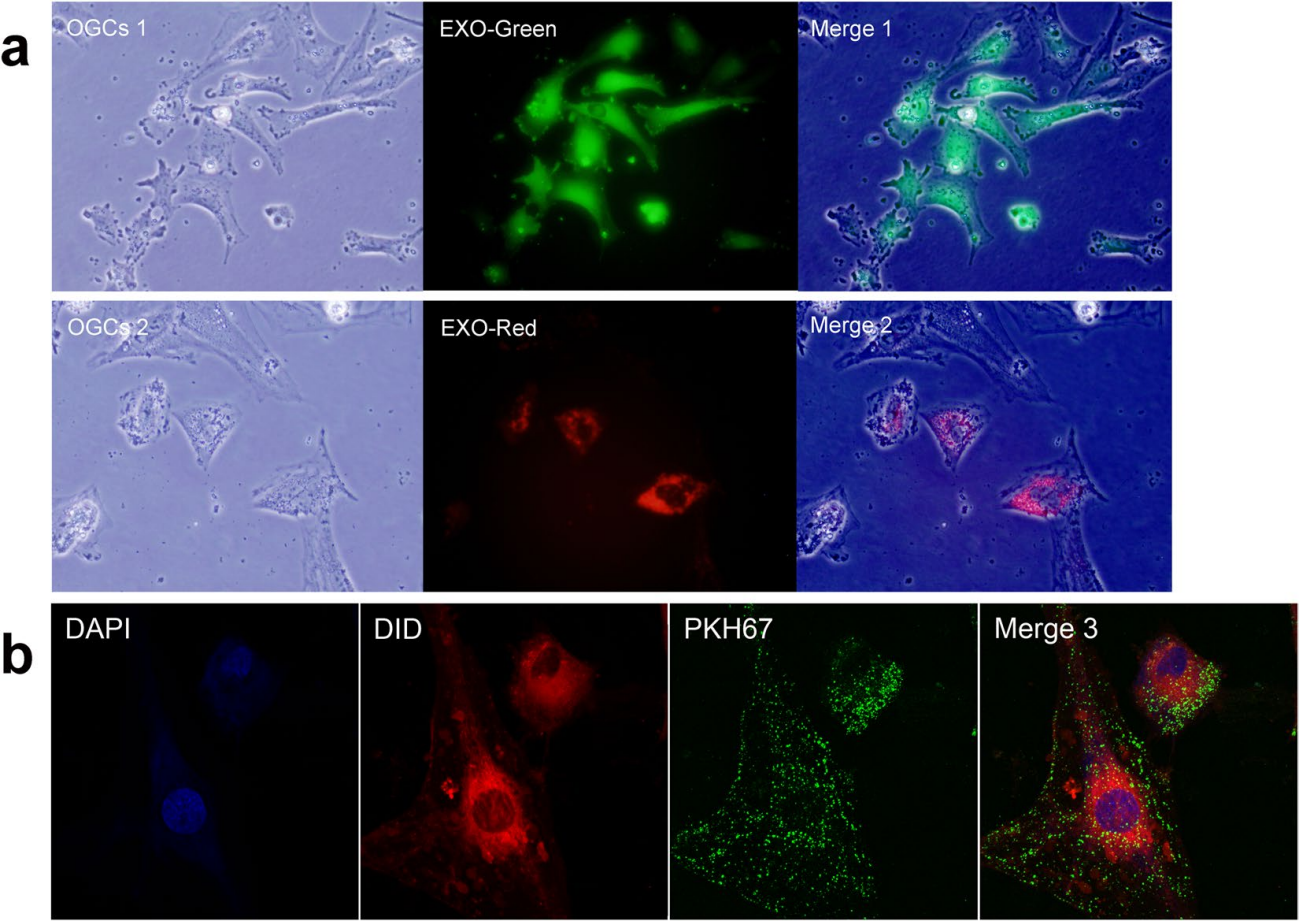
Uptake of huMSC-EXOs by OGCs. (**a**) The protein component of Exo-Green-labelled huMSC-EXOs (100 µg, 100 µg/ml) gathered in the interior of the cells. Similarly, microRNAs in Exo-Red-labelled huMSC-EXOs entered into the cells (×400 magnification). (**b**) HuMSC-EXOs (PKH67/green, 100 µg, 100 µg/ml) are adsorbed on the surface of OGCs (DID/red) or engulfed in the OGCs after image overlay (×630 magnification).

Using fluorescence microscopy, the protein component of huMSC-EXOs, which was labelled with the fluorescent reagent Exo-Green, could be observed by monitoring the green fluorescence, and the green fluorescence gathered in the interior of the cells. Similarly, microRNAs in huMSC-EXOs labelled with the fluorescent reagent Exo-Red could be seen as red fluorescence. The cells could effectively combine with huMSC-EXOs dyed with Exo-Green and Exo-Red (Fig. 4a).

Another fluorescent labelling system in which huMSC-EXOs, the OGC membrane and nuclei were labelled with PKH67, DID and DAPI, showed green, red, and blue fluorescence, respectively, under confocal microscopy. After merging the images, we found that huMSC-EXOs (green) had been adsorbed or engulfed by the OGCs (red), indicating that huMSC-EXOs successfully entered the OGCs (Fig. 4b).

**Figure 5:**
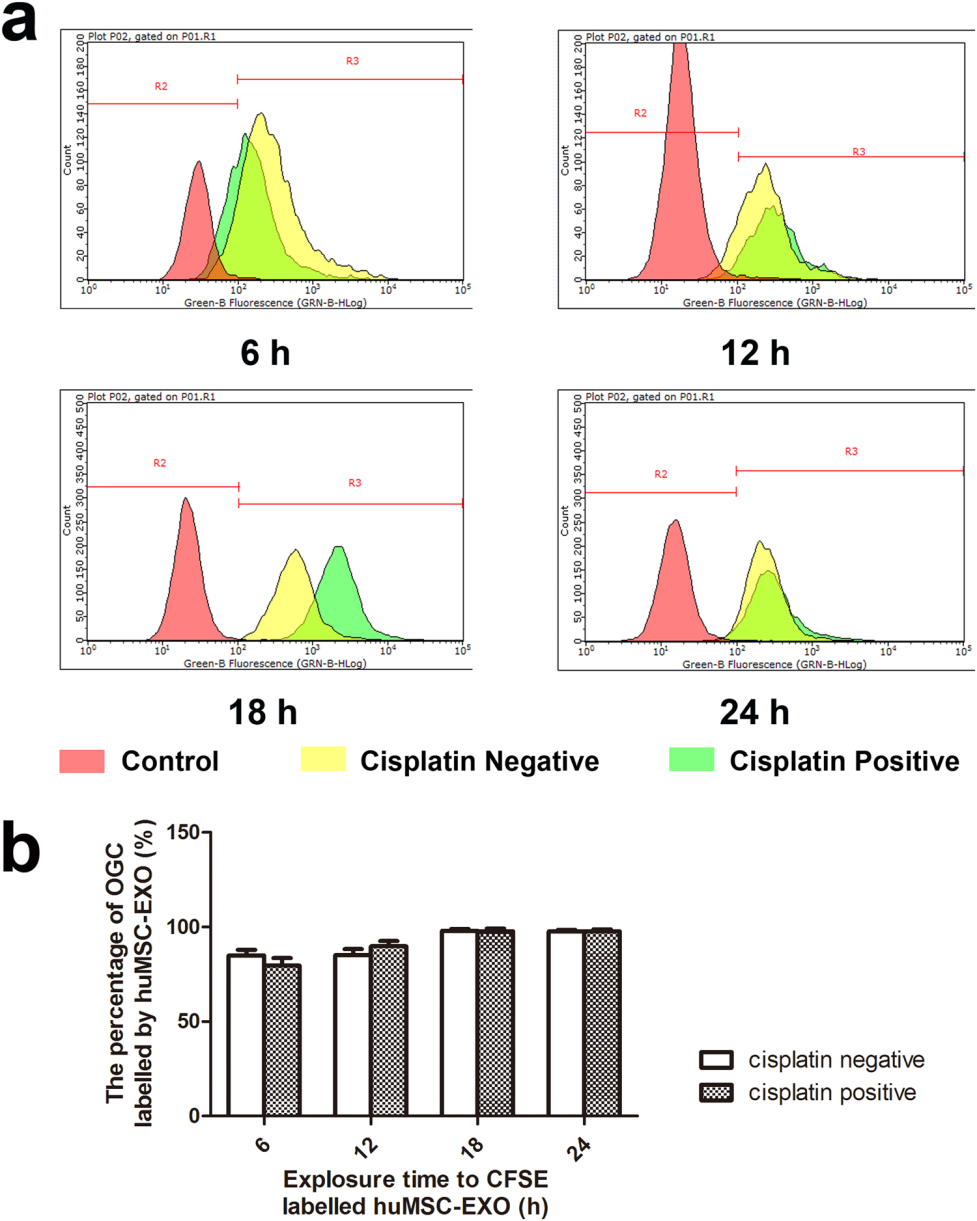
Quantitative uptake ratio of huMSC-EXOs and the effect of cisplatin. (**a**,**b**) In the cisplatin-negative group, the percentage of huMSC-EXO-labelled cells, analysed with guavaSoft 3.1.1, was 84.93 ± 5.23%, 85.19 ± 5.37%, 98.06 ± 1.48% and 97.67 ± 1.51% at 6 h, 12 h, 18 h and 24 h, respectively, whilst the percentage was 79.56 ± 7.00%, 89.83 ± 4.73%, 97.81 ± 2.49% and 97.68 ± 1.89%, respectively, in the cisplatin-positive group. The results showed that huMSC-EXOs were effectively taken up by OGCs, and the uptake ratio was not different in the presence of 4 µg/ml cisplatin (P < 0.05); n = 3.

Carboxyfluorescein diacetate succinimidyl ester (CFSE) was used to label huMSC-EXOs to quantitatively determine the uptake ratio. The detection indicator was the percentage of cells with bound CSFE-labelled huMSC-EXOs, which reflected the uptake ratio of huMSC-EXOs. In the cisplatin-negative group, the percentages of the huMSC-EXO-labelled cells analysed by guavaSoft 3.1.1 were 84.93 ± 5.23%, 85.19 ± 5.37%, 98.06 ± 1.48% and 97.67 ± 1.51% at 6 h, 12 h, 18 h and 24 h, respectively, whilst the percentages were 79.56 ± 7.00%, 89.83 ± 4.73%, 97.81 ± 2.49% and 97.68 ± 1.89%, respectively, in the cisplatin-positive group. The results showed that huMSC-EXOs were effectively taken up by OGCs, and the uptake ratio was not different when 4 µg/ml of cisplatin was used in the experiments (P < 0.05); n = 3 (Fig. 5a and b).

All of the above-mentioned observations verified that huMSC-EXOs can effectively bind OGCs, and the uptake ratio was not different when 4 µg/ml cisplatin was used in the experiments, which produced a foundation of biological behaviours for the following experiments.

### HuMSC-EXOs protect OGCs from cisplatin-induced injury and promote resistance to cell apoptosis *in vitro*

**Figure 6:**
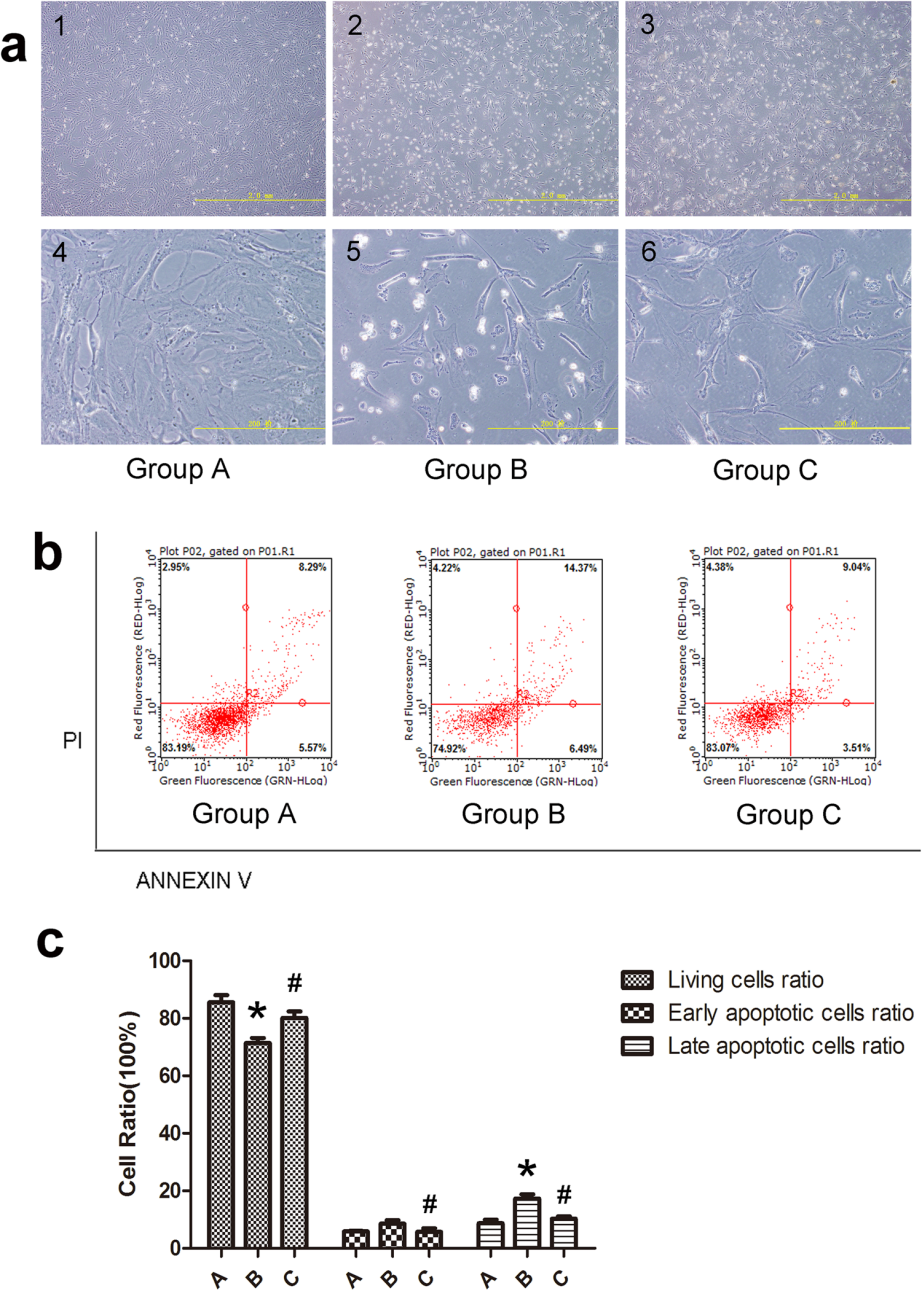
HuMSC-EXOs protect against cisplatin-induced injury of OGCs and promote resistance to cell apoptosis *in vitro* based on FACS. (**a**) Groups A, B and C were cultured for 48 h, and the number of apoptotic cells in group B was higher than that in group C under the microscope (a1–a3: ×40 magnification; a4–a6: ×400 magnification). (**b**,**c**) Through annexin-V-FITC/PI double staining and FACS analysis, the proportion of living cells between groups A and B was found to be different (P < 0.05). Similarly, the proportion of living cells in group B compared with group C was also different (P < 0.05). No significant difference (P > 0.05) in the percentage of early apoptotic cells between groups A and B was observed, whilst in groups B and C, a difference was observed (P < 0.05). For the percentage of late apoptotic cells, a difference was observed between group A and group B (P < 0.05), along with groups B and C (P < 0.05); n = 5. *Group B vs. group A. ^#^Group C vs. group B.

OGCs cultured in six-well plates were divided into 3 groups: group A (control group), group B (cisplatin injury group) and group C (huMSC-EXO coculture group). After 48 h, the cells in groups A, B and C were observed under a microscope, and the apoptosis in group B was found to be higher than that in group C (Fig. 6a). As determined by annexin-V-FITC/PI staining and FACS analysis, the percentages of living cells, early apoptotic cells and late apoptotic cells in group A were 85.50 ± 4.45%, 5.86 ± 0.50% and 8.69 ± 2.15%, respectively. The percentages in group B were 71.37 ± 3.10%, 8.58 ± 2.04% and 17.26 ± 2.67%, respectively, and the percentages in group C were 80.09 ± 4.00%, 5.72 ± 2.15% and 10.27 ± 1.46%, respectively. The proportion of living cells between groups A and B was significantly different (P < 0.05). And the percentages of living cells in group C when compared with group B was also different (P < 0.05). No significant difference (P > 0.05) in the percentage of early apoptotic cells between groups A and B was observed, whilst group B and group C were different (P < 0.05). For the percentage of late apoptotic cells, group A was different from group B (P < 0.05), as were groups B and C (P < 0.05); n = 5 (Fig. 6b and c).

**Figure 7:**
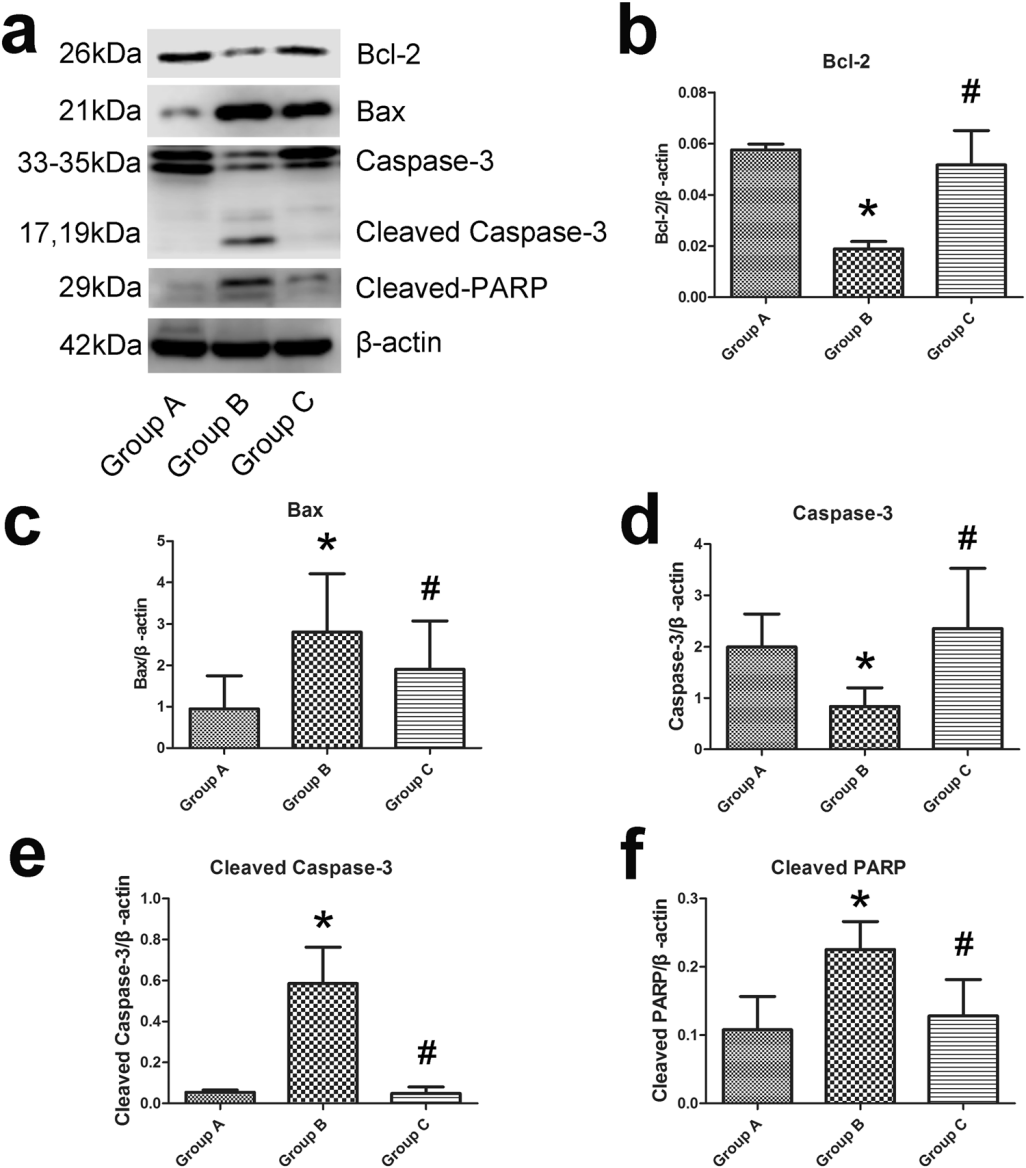
HuMSC-EXOs protect against cisplatin-induced injury of OGCs and promote resistance to cell apoptosis *in vitro* based on western blotting. (**a**) Western blotting revealed significant differences (P < 0.05) between groups A and B in the levels of Bax, cleaved caspase-3, Bcl-2 and cleaved PARP. (**b**–**f**) The expression of Bax, cleaved caspase-3 and cleaved PARP in group B was increased compared with that in group A, whilst the expression of Bcl-2 was decreased. However, the expression of Bax, cleaved caspase-3 and cleaved PARP in group C was reduced compared with that in group B, and the expression of Bcl-2 was increased; n = 5. *Group B vs. group A. ^#^Group C vs. group B.

Western blotting was used to detect changes in the expression of apoptosis-related proteins and DNA repair proteins, and the expression of Bax, cleaved caspase-3, Bcl-2 and cleaved PARP was found to be significantly different (P < 0.05) between groups A and B and groups B and C. The expression of Bax, cleaved caspase-3 and cleaved PARP in group B was increased compared with group A, whilst that of Bcl-2 was decreased. However, the expression of Bax, cleaved caspase-3 and cleaved PARP in group C was reduced compared with group B, and Bcl-2 expression was increased; n = 5 (Fig. 7).

Based on the regulation of apoptosis-related proteins, huMSC-EXOs had a robust protective effect on the cisplatin-induced damage of OGCs. Precise regulation of apoptosis was achieved by exosomes, perhaps via specific microRNAs that modified certain genes or proteins. However, cell apoptosis is a complex multi-pathway process, and thus, the effect of the exosomes on apoptosis may be not as dramatically represented in the results of the annexin-V/PI staining and FACS analysis as in the western blotting results.

### Predicted target genes of microRNAs determined with a PCR array

The results indicated that microRNAs with high abundance existed in huMSC-EXOs, and some of the predicted target genes were listed to provide further evidences that these micro-RNAs had a relationship with OGC apoptosis and could participate in regulation of the apoptotic process (Table 1). U6 was considered the internal reference in the PCR assay. If the *∆Ct* value was lower, the expression level of microRNAs in the exosomes was higher. We used databases (mirBase and TargetScan) to predict and analyse the potential target genes of the microRNAs with high abundance in Table 1, which we expect will be helpful in our future studies. The results predicted that microRNA-24, microRNA-106a, microRNA-19b and microRNA-25 may be closely related to apoptosis; n = 3.

**Table 1:** Predicted target genes analysis of microRNAs expressed in huMSC-EXOs.

MicroRNAs	*∆Ct* (N = 3)	Predicted target genes (mirBase and TargetScan)	Related diseases and references
	Means ± SD		
UniSp6 (U6)	20.67 ± 2.35	N/A	N/A
microRNA-125b-5p	21.47 ± 1.40	DNA-damage regulated autophagy modulator 2 (**DRAM2**) BCL2-antagonist/killer 1 (**BAK1**)	Retinoblastoma^37^; Alzheimer’s disease^38^; Chronic myeloid leukemia^39^
microRNA-21-5p	21.64 ± 1.95	Interleukin 12 A (**IL12A**) Fas ligand (TNF superfamily, member 6) (**FASLG**) Cchemokine (C-C motif) ligand 1 (**CCL1**) Pleckstrin homology domain containing, family A (phosphoinositide binding specific) member 1 (**PLEKHA1**)	Asthmatic^40^; Autoimmune lymphoproliferative syndrome^41^
microRNA-24-3p	22.38 ± 1.73	BCL2-like 11 (apoptosis facilitator) (**BCL2L11**) Topoisomerase (DNA) I (**TOP1**) Fascin homolog 1 (**FSCN1**)	Gastric cancer^42^; Senescence^43^; nasopharyngeal carcinoma^44^
microRNA-16-5p	23.50 ± 1.34	Cyclin E1 (**CCNE1**) RAB23, member RAS oncogene family (**RAB23**)	Cervical cancer^45^; Inflammatory Pain^46^
microRNA-92a-3p	23.94 ± 0.81	F-box and WD repeat domain containing 7, E3 ubiquitin protein ligase (**FBXW7**) RAB23, member RAS oncogene family (**RAB23**)	Osteosarcoma^47^
microRNA-100-5p	23.98 ± 0.99	Bromodomain adjacent to zinc finger domain, 2 A (**BAZ2A**)	Prostate cancer^48^
microRNA-106a-5p	24.81 ± 1.34	Autophagy-related gene 7 (**ATG7**) Mitofusin 2 (**MFN2**) Phosphatase and tensin homologue (**PTEN**) Programmed cell death 4 (**PDCD4**) C-Jun NH2-terminal kinase/mitogen activated kinase-like protein (**JNK/MAPK**)	Colorectal cancer^49^; Osteosarcoma^50^; Glioma^51^; Ovarian cancer^32^
microRNA-19b-3p	24.85 ± 1.42	Zinc finger, MYND-type containing 11 (**ZMYND11**) Actin filament associated protein 1/caspase7 (**AFAP1 and CASE7**) Ring finger protein 11 (**RNF11**)	Coronary artery disease^34^; Encephalitis Virus-Mediated Inflammation^52^
microRNA-145-5p	24.95 ± 1.48	Fascin homolog 1 (**FSCN1**) Myosin VA (**MYO5A**)	Metastatic melanoma^53^
microRNA-25-3p	25.50 ± 0.80	Cyclin E1 (**CCNE1**)	Human Gastric Adenocarcinoma^35^
microRNA-let-7a-5p	25.56 ± 0.48	Tripartite motif containing 71 (**TRIM71**)	Embryonic neural tube disease^54^

## Discussion

Studies have previously demonstrated that huMSCs can be used to treat POI and protect OGCs from damage by cisplatin^15^, but the exact mechanisms responsible for this protection are unclear. Therefore, we considered whether the effects of huMSCs on OGCs occurred via a paracrine secretion mechanism. In this study, we determined whether exosomes derived from huMSCs had the same therapeutic or protective effects on cisplatin-induced OGCs damage and explored the preliminary mechanisms of these effects. We demonstrated that huMSC-EXOs ameliorated cisplatin-induced OGC stress and apoptosis *in vitro*, which provides a cytological basis for subsequent experiments *in vivo*.

The cellular lipid bilayer retracts to form multi-chambered vesicles, which release nanoscale vesicles (exosomes) in a calcium-dependent manner that bind to cell membranes. Exosomes are 30 to 200 nm in size, spherical in shape, and mediate local cell-to-cell communication by transferring mRNA, lncRNA, microRNA, proteins and lipids^17^. It has been reported that CD9, CD63, Hsp70 and CD81 are frequently located on the surface of exosomes, whilst the endoplasmic reticulum marker calnexin and lysosome marker Lamp 1 are typically not present. Target cells can absorb exosomes in several ways, including membrane fusion, endocytosis and receptor binding^22^. Previous studies have shown that huMSC-EXOs promote tissue injury repair through horizontal transfer of proteins and microRNAs^27, 28^. The exosomes obtained from huMSCs in this study had the same characteristics: they exhibited spheroid morphology, and TEM confirmed sizes of 30 to 200 nm. Western blotting analyses showed that the exosomes were positive for CD9, CD63, Hsp70 and CD81 expression and negative for calnexin and Lamp 1. Hence, they were used in subsequent experiments.

In the present study, we successfully cultured huMSCs and rat OGCs, isolated exosomes derived from huMSCs and confirmed that huMSC-EXOs contain a variety of microRNAs. Moreover, by establishing an injury model *in vitro*, it was observed that huMSC-EXOs could be incorporated into injured OGCs, thus accelerating the recovery of OGCs from the stress and apoptosis induced by cisplatin *in vitro*.

First, the effective uptake of exosomes by cells was the basis for the subsequent biological effects, and two different fluorescent labelling methods were used to observe this behaviour via fluorescence microscopy and confocal microscopy. After administration of huMSC-EXOs, the level of OGC apoptosis was limited, and the number of apoptotic cells was reduced compared to the cisplatin group (group B). When cells are subjected to oxidative stress, the level of proapoptotic proteins increases to inhibit the activity of antiapoptotic proteins, which can decrease mitochondrial activity and initiate apoptosis. It has been suggested that cisplatin-induced cell damage was associated with a rise in the level of the proapoptotic protein Bax and a reduction in the antiapoptotic protein Bcl-2. In this study, the huMSC-EXO group (group C) exhibited an evident decrease in Bax expression compared to the cisplatin alone treatment group (group B), whilst the Bcl-2 protein level was increased when huMSC-EXOs were present *in vitro*. In addition, cleaved caspase-3, as the executor of apoptosis, was highly expressed in the cisplatin group, whilst expression was decreased in the huMSC-EXO group. Moreover, PARP is a poly(ADP-ribose) polymerase and a DNA repair-related protein, which can be used as a substrate of caspase-3 for degradation. If the level of cleaved PARP increases, then DNA damage is severe and DNA breaks are complex, resulting in a variety of further cascades that induce apoptosis^29^. In our results, it was found that the huMSC-EXO group had an evident reduction in cleaved PARP compared to the cisplatin group. Although cisplatin has been reported to affect endocytosis of proteins^30^, it was shown through supplementary experiments that huMSC-EXOs can effectively bind to OGCs, and the uptake ratio had no significant relationship with the existence of 4 µg/ml cisplatin in our study.

However, the effects on apoptosis observed in the annexin-V/PI staining and FACS results were not as dramatic as those revealed by the western blotting results. Exosomes carry a variety of microRNAs and proteins into target cells and precisely regulate or modify certain genes or proteins, whilst cell apoptosis is a complex multi-pathway process. The expression of one of the apoptosis-related proteins changed, which indicated that the components of exosomes may regulate apoptosis-related genes or mRNAs, either directly or indirectly, but the apoptotic process also involves many other pathways and regulatory mechanisms, coupled with the repair mechanism of the cells, resulting that the final effect of exosomes on cells would likely not show up as a very significant antiapoptotic effect.

A variety of microRNAs were observed in huMSC-EXOs, and a qRT-PCR array analysis of huMSC-EXOs was conducted. MicroRNAs are a series of small noncoding RNAs (~22 nucleotides long) that regulate the expression of target genes at the post-transcriptional level. During this process, the microRNA/microRNA-induced silencing complex (miRISC) binds the 3′-UTR of target mRNA to inhibit expression via translational repression and/or mRNA degradation^24^. Databases (mirBase and TargetScan) were used to predict and analyse the potential targets of the microRNAs with high abundance in Table 1, which was expected to be helpful for subsequent studies. We predicted that microRNA-24, microRNA-106a, microRNA-19b and microRNA-25 may be closely related to apoptosis. Sang *et al*. identified microRNAs present in microvesicles and the supernatant of human follicular fluid, and microRNA-24 was found to regulate oestradiol concentrations and progesterone concentrations, which shows that the highly expressed microRNA-24 targets genes associated with reproductive, endocrine, and metabolic processes^31^. MicroRNA-106a is also closely related to ovarian development; some studies have shown that downregulation of the expression of microRNA-106a inhibits cell growth and metastasis of ovarian cancer cells^32^. In addition, ovarian microcirculation density reflects ovarian function, and human amnion epithelial cell treatment enhances angiogenesis primarily through paracrine pathways in the ovaries^33^. Meanwhile, Tang *et al*. demonstrated that microRNA-19b plays a key role in attenuation of TNF-α-induced endothelial cell apoptosis and that this function is closely linked to the Apaf1/caspase-dependent pathway, and therefore, it can be speculated that elevated microRNA-19b may be beneficial for restoring ovarian function by increasing the antiapoptotic ability of vascular endothelial cells^34^. Moreover, microRNA-25 has an antiapoptotic role in human gastric adenocarcinoma cells, possibly via inhibition of FBXW7, thus promoting the expression of oncogenes such as CCNE1 and MYC^35^. All of these forward-looking, predictive and instructive results could provide background and ideas for our subsequent studies on microRNAs derived from huMSC-EXOs.

Hence, we cultured huMSCs and rat OGCs and successfully isolated exosomes derived from huMSCs. In addition, we observed that huMSC-EXOs could become incorporated into injured OGCs, thus accelerating the recovery of OGCs after stress and apoptosis induced by cisplatin *in vitro*.

Next, we will focus on *in vivo* experiments and the protection mechanism of microRNAs contained in exosomes secreted by huMSCs in protection. In this study, the precise mechanism of how huMSC-EXOs protect cisplatin-induced OGC damage is still unclear, but it can be concluded from the results of the present study that huMSC-EXOs can promote resistance to cisplatin-induced OGC apoptosis and protect OGCs from cisplatin-induced injury *in vitro*.

## Methods

The experiments were conducted in accordance with approved guidelines: the animal experiments were performed according to the Guide for the Care and Use of Laboratory Animals. The experimental protocols were approved by the Institutional Review Board of Qilu Hospital, Shandong University (No. KYLL-2015(KS)-077), and informed consent was obtained from all patients before the study.

### Isolation and characterization of huMSCs

HuMSCs were isolated and cultured according to methods previously described^5, 36^. HuMSCs were cultured in α-MEM (HyClone, Logan, UT, USA) containing 10% foetal bovine serum (FBS, Life Technologies, USA) and 1% penicillin and streptomycin (100×, Life Technologies) at 37 °C, 5% CO_2_ and 100% H_2_O.

To observe morphology, adherent cells were stained with Wright’s stain and imaged with a JEOL-1200EX transmission electron microscope, and images were recorded with a MORADA-G2 camera.

To detect typical surface markers of huMSCs, FACS was performed using the following phycoerythrin (PE)-conjugated, fluorescein isothiocyanate (FITC)-conjugated, percp-Cy5.5-conjugated or allophycocyanin (APC)-conjugated mouse antihuman monoclonal antibodies: PE-CD29, PE-CD31, PE-CD34, FITC-CD44, PE-CD45, PE-CD73, PerCp-Cy5.5-CD90, PE-CD105, PE-CD271, APC-CD133, PE-HLA-DR and PE-HLA-ABC (BD Biosciences, Franklin Lakes, NJ, USA). HuMSCs were collected in 100 µl of PBS. Every tube contained 1 × 10^5^ cells, and antibodies were added. After incubation at room temperature for 20 min, the cells were examined with a Guava easyCyte HT flow cytometer (Millipore, Billerica, MA, USA). The results were analysed with guavaSoft 3.1.1 software; n = 5.

### Isolation and characterization of exosomes derived from huMSCs

HuMSCs were cultured in BioWhittaker ultraCULTURE general purpose serum-free medium (Lonza, Basel, Switzerland) containing 2% Ultroser G serum substitute (Pall, Port Washington, NY, USA), referred to hereafter as serum-free medium. The medium was collected after 48 h. The medium was processed by 400 g centrifugation for 15 min and by 10,000 g centrifugation for 15 min at 4 °C. The supernatant was further filtered using a 0.22 µm filter (Millipore) and eventually ultracentrifuged at 100,000 g for 5 h at 4 °C. The exosome pellets were resuspended in PBS and stored at −80 °C for further use. The concentration of exosomal protein was quantified using a Pierce BCA Protein Assay Kit (Thermo Scientific, Rockford, IL, USA).

The morphology of the exosomes was observed using TEM. Exosomes were identified by specific antibodies for CD63 (Abcam, Cambridge, UK), CD9 (Abcam), CD81 (System Biosciences, Palo Alto, CA, USA), heat shock protein 70 (Hsp70, System Biosciences), calnexin (Cell Signaling Technology, Danvers, MA, USA) and Lamp 1 (Cell Signaling Technology), which were used for western blotting analysis. All of these antibodies dilution ratios were 1:1,000; n = 5.

### Isolation and characterization of rat OGCs and establishment of a cell model

Healthy female Wistar rats, weighing 50–60 g, were chosen as experimental animals. Each rat was subcutaneously injected with 40 IU of FSH (Solarbio, Beijing, China). Rats were sacrificed after 48 h. Both ovaries were collected, and the surrounding fat and fascia were removed. Then, ovarian tissues were cut into cubes less than 1 cm^3^, digested with 0.25% trypsin (containing 0.01% EDTA, Life Technologies) at 37 °C for 60 min and shaken once every 10 min. The cell suspension was filtered with a 70 µm mesh filter and centrifuged at 1,000 r/min for 10 min. The collected cells were washed once with PBS and cultured in DMEM/F12 = 1:1 (HyClone) containing 10% FBS and 1% penicillin and streptomycin at 37 °C, 5% CO_2_ and 100% H_2_O. Each well was inoculated with 2 × 10^5^ cells in the six-well, and we chose OGCs that adhered about 50–60% plate into the subsequent experiments.

The FSHR is a specific marker of OGCs, and thus, OGCs were identified using immunohistochemistry with an FSHR rabbit antimouse polyclonal antibody (Boster, Wuhan, China). OGCs were cultured on glass coverslips. The cells were fixed in cold acetone for 15 min, immersed in 3% H_2_O_2_ for 10 min at room temperature and subsequently washed 3 times with PBS. The cells were blocked in goat serum for 20 min at room temperature and then incubated with primary antibodies (dilution ratio = 1:200) overnight at 4 °C. Afterwards, the cells were washed 3 times with PBS and incubated for 70 min at 37 °C with horseradish peroxidase (HRP)-labelled goat anti-rabbit IgG (dilution ratio = 1:1,000, ZSGB-BIO, Beijing, China) and HRP solution. Finally, the cells were dyed with 3,3′-diaminobenzidine (DAB) and counterstained with haematoxylin (Solarbio). Images were acquired with a microscope; n = 5.

Cisplatin (Qilu Pharmaceutical, Jinan, China) was used to establish a cell model. Based on our pre-test results, 4 µg/ml cisplatin was chosen for the following experiments.

### Uptake of huMSC-EXOs by OGCs visualized with fluorescent labelling

#### Fluorescence microscopy

The fluorescent reagent Exo-Green (System Biosciences) was used to label the protein component of huMSC-EXOs, and Exo-Red (System Biosciences) was used to label the microRNAs in huMSC-EXOs. HuMSC-EXOs were labelled with Exo-Green and Exo-Red, separately, for 20 min at 37 °C, and then, the labelled huMSC-EXOs were washed with PBS and re-pelleted twice using ExoQuick-TC exosome precipitation solution (System Biosciences). Exo-Green-labelled huMSC-EXOs (100 µg, 100 µg/ml) were incubated with OGCs in a six-well plate for 2 h at 37 °C, whilst Exo-Red-labelled huMSC-EXOs were incubated for 24 h. Images were acquired with a fluorescence microscope.

#### Confocal Microscopy

A huMSC-EXO suspension was labelled with PKH67 [green] (PKH67 Fluorescent Cell Linker Kit, Sigma-Aldrich, St. Louis, MO, USA) for 5 min, and the reaction was stopped by the addition of exosome-depleted FBS. Then, huMSC-EXOs were washed with PBS and re-pelleted twice using ExoQuick-TC exosome precipitation solution. The cells were cultured on glass coverslips in a six-well plate. OGCs were incubated with PKH67-labelled huMSC-EXOs (100 µg, 100 µg/ml) for 5 h and then dyed in medium containing 5 µM 1,1′-dioctadecyl-3,3,3,3-tetramethylindodicarbocyanine (DID [red]) (Biotium, Fremont, CA, USA) at 37 °C for 20 min. After fixation with 4% paraformaldehyde for 20 min, the cells were mounted with Fluoroshield mounting medium with 4′,6-diamidino-2-phenylindole (DAPI [blue]) (Abcam). Pictures were obtained with a confocal laser scanning microscope using ZEN software (Carl Zeiss, German).

#### Quantitative uptake ratio of huMSC-EXOs and the effect of cisplatin

CFSE (Life Technologies) was used to label huMSC-EXOs. HuMSC-EXOs were suspended in PBS and incubated for 20 min at 37 °C in 5 µM CFSE. The CFSE-labelled huMSC-EXOs were washed with PBS and re-pelleted twice using ExoQuick-TC exosome precipitation solution to remove any free dye remaining in the solution. Afterwards, huMSC-EXOs (100 µg, 100 µg/ml) were resuspended in either cisplatin-negative or cisplatin-positive serum-free medium and cultured with OGCs of each well. The cisplatin concentration was 4 µg/ml. The cells were collected at 6 h, 12 h, 18 h and 24 h and analysed with flow cytometry. The detection indicator was the percentage of cells bound with CSFE-labelled huMSC-EXOs, which reflected the uptake ratio of huMSC-EXOs. The results were analysed by guavaSoft 3.1.1 software; n = 3.

### The effect of huMSC-EXOs on cisplatin-damaged OGCs

We chose OGCs that adhered about 50–60% in the six-well plate into the subsequent experiments. OGCs cultured in six-well plates were divided into 3 groups: group A (blank control group), group B (cisplatin injury group) and group C (huMSC-EXO coculture group). Cisplatin and huMSC-EXOs were added to group C at the same time. The working concentration of cisplatin was 4 µg/ml. HuMSC-EXOs (100 µg, 100 µg/ml) were added to each well, and the plates were cultured for 48 h. The cells were collected for the following analyses.

#### FACS analysis

Annexin-V and propidium iodide staining (Annexin V-FITC Apoptosis Detection Kit; BD Biosciences) were used to analyse the percentage of apoptotic cells. The experimental process was followed by the manufacturer’s instruction. The results were obtained with flow cytometry and analysed with guavaSoft 3.1.1 software; n = 5.

#### Western blotting analysis

OGCs in different groups were collected and lysed in radioimmunoprecipitation assay (RIPA) buffer (Solarbio) containing 1 mM phenylmethanesulfonyl fluoride (PMSF) (Solarbio) at 4 °C for 30 min. The protein concentration was determined with a BCA Protein Assay Kit. Up to 50 µg of protein was electrophoresed on 12% SDS-polyacrylamide gels and transferred onto polyvinylidene difluoride (PVDF) membranes (Millipore), which were blocked with 5% nonfat dry milk (BD Biosciences) for 1 h at room temperature. The membranes were then blotted with primary antibodies at 4 °C overnight. The following primary antibodies were used: β-actin (1:1,000, Proteintech, Rosemont, IL, USA), B cell lymphoma 2 protein (Bcl-2, 1:1,000, Cell Signaling Technology), Bcl-2-associated X protein (Bax, 1:2,000, Abcam), caspase-3 (1:1000, Cell Signaling Technology), cleaved caspase-3 (1:1,000, Cell Signaling Technology) and cleaved poly-ADP-ribose polymerase (cleaved PARP, 1:250, Abcam). Then, the PVDF membrane was washed 3 times with Tris-buffered saline/Tween (Solarbio) and incubated with HRP-conjugated secondary antibody (1:10,000, ZSGB-BIO) for 70 min at room temperature. Detection was performed using Luminata western HRP substrate (Millipore). The results were obtained with a LI-COR 3600 instrument (LI-COR Biosciences, Lincoln, NE, USA) and analysed with an Image Studio Digits Version 4.0 system; n = 5.

### HuMSC-EXO microRNA array (qRT-PCR analysis)

Total RNA was isolated from huMSC-EXOs using TRIzol reagent (Life Technologies). Two microgram aliquots of RNA were synthesized according to the manufacturer’s protocol (Vazyme, Nanjing, China). cDNAs were synthesized using 5× Reaction buffer (Exiqon, Vedbaek, Denmark) and Enzyme mix (Exiqon). Array analyses were performed using microRNA PCR arrays (SYBR Green master mix) (Exiqon) on an ABI PRISM7900 system (Applied Biosystems, Rockford, IL, USA); n = 3.

### Statistical analysis

Data are expressed as the means ± SD. Data were analysed using one-way ANOVA or Student’s t-test. Statistical analysis was performed using GraphPad Prism 5 (GraphPad Software, Inc., La Jolla, CA). Images were processed using Photoshop CS5 V12.0.1. A value of P < 0.05 was considered statistically significant.
